# Yield of household contact tracing for tuberculosis in rural South Africa

**DOI:** 10.1186/s12879-018-3193-7

**Published:** 2018-07-04

**Authors:** Kristen M. Little, Reginah Msandiwa, Neil Martinson, Jonathan Golub, Richard Chaisson, David Dowdy

**Affiliations:** 1 Department of Epidemiology, Johns Hopkins Bloomberg School of Public Health, 615 N Wolfe St, Baltimore, MD 21205 USA; 2 Perinatal HIV Research Unit, Nurses Residence, Chris Hani Baragwanath Academic Hospital, Soweto, South Africa; 3 Johns Hopkins University School of Medicine, Baltimore, MD USA

**Keywords:** Tuberculosis, Rural, Active case finding

## Abstract

**Background:**

Efficient and effective strategies for identifying cases of active tuberculosis (TB) in rural sub-Saharan Africa are lacking. Household contact tracing offers a potential approach to diagnose more TB cases, and to do so earlier in the disease course.

**Methods:**

Adults newly diagnosed with active TB were recruited from public clinics in Vhembe District, South Africa. Study staff visited index case households and collected sputum specimens for TB testing via smear microscopy and culture. We calculated the yield and the number of households needed to screen (NHNS) to find one additional case. Predictors of new TB among household contacts were evaluated using multilevel logistic regression.

**Results:**

We recruited 130 index cases and 282 household contacts. We identified 11 previously undiagnosed cases of bacteriologically-confirmed TB, giving a prevalence of 3.9% (95% CI: 2.0–6.9%) among contacts, a yield of 8.5 per 100 (95% CI: 4.2–15.1) index cases traced, and NHNS of 12 (95% CI: 7–24). The majority of new TB cases (10/11, 90.9%) were smear negative, culture positive. The presence of TB symptoms was not associated with an increased odds of active TB (aOR: 0.3, 95% CI: 0.1–1.4).

**Conclusions:**

Household contacts of recently diagnosed TB patients in rural South Africa have high prevalence of TB and can be feasibly detected through contact tracing, but more sensitive tests than sputum smear are required. Symptom screening among household contacts had low sensitivity and specificity for active TB in this study.

## Background

More aggressive approaches to finding cases are essential if we are to accelerate the decline in TB incidence [1]. While most TB control interventions have focused largely on urban, high-burden settings, high TB incidence rates have been observed in rural populations [2, 3], where long distances [4], inadequate infrastructure, poor-quality health facilities, and limited human resources present major obstacles to active case finding efforts [5, 6]. To date, the majority of research on active TB case finding has been done in urban, peri-urban, or congregate settings [7]; limited research has been done on the efficacy and feasibility of these interventions in rural areas [8].

Though unproven, it is reasonable to believe that a higher proportion of TB transmission could occur in the household in rural settings, where fewer people may frequent high-transmission settings such as public transit, shebeens, or major public gatherings [9–11]. We therefore sought to determine the yield of a household-based active case-finding intervention in a rural region of South Africa, a country with high rates of both TB and HIV [12]. We aimed to estimate the prevalence of previously undiagnosed TB among household contacts of recently diagnosed adult TB patients, to calculate the number of households needed to screen (NHNS) to identify one additional case of previously undiagnosed TB, and to qualitatively compare the yield, prevalence, and NHNS of active TB case finding in this rural setting to a higher-burden, peri-urban one [13].

## Methods

### Study setting

This study took place in Vhembe District, a municipality in Limpopo Province, South Africa, that borders Zimbabwe and Botswana. The district has a population of approximately 1.3 million, and a population density of 130 persons per square mile [14]. At 350 per 100,000/year, Vhembe had the second lowest district-level TB incidence in South Africa in 2012 [15].

### Participants

**Fig. 1:**
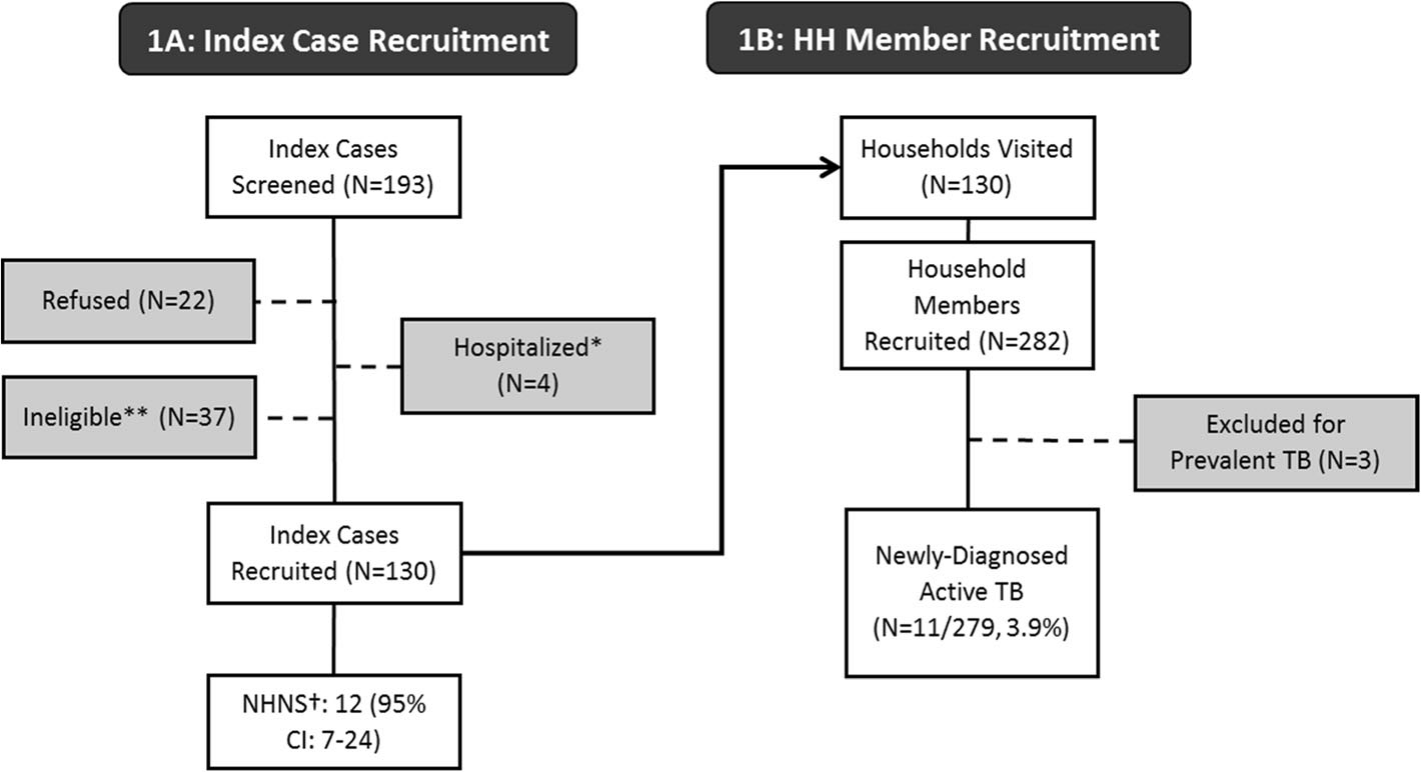
Index client recruitment is illustrated in Panel **A**, and household member recruitment in Panel **B**. ** Index participants were ineligible due to age < 18 years (*N* = 1), a time between TB treatment initiation and study screening of > 30 days (*N* = 5), having no household contacts (*N* = 22), or primary residence outside of the study district (*N* = 9). ^†^ NHNS: Number of (index case) households needed to screen occupants with smear and culture to find 1 new case of active TB among household contacts

Adults recently diagnosed with TB at public clinics in the district (“index cases”) were consecutively asked to participate in the study. Index cases were eligible to participate if they were ≥ 18 years old, had a recorded TB diagnosis based on clinical evaluation and/or radiology (with or without bacteriological confirmation), had initiated TB treatment within the previous 30 days, had been a resident of Vhembe District for at least 6 months, had at least one household contact, and consented to a home visit by the study team (Fig. 1). A household contact was defined as any person living on the same residential plot who shared either the same residential structure or frequent meals with the index case. Participating index cases provided written informed consent and completed a survey that included demographics, TB and HIV clinical history, and directions to their home. TB diagnosis and treatment data were abstracted from the clinic registers and/or patient’s clinical records.

### Household visits

Study staff visited index case households within 2 weeks of recruitment. Household contacts were eligible to participate in the study if they met the definition of a household contact and provided informed consent. Trained study staff administered similar brief surveys to all participating household contacts, and collected one sputum specimen from each respondent for smear and culture in accordance with national guidelines [16]. Sputum was induced when expectoration was not possible. TB testing, including fluorescence microscopy with auramine staining and culture in liquid media (Mycobacteria Growth Indicator Tube [MGIT] 960, BD Diagnostics, Franklin Lakes, USA) was performed by South Africa’s National Health Laboratory System. Positive cultures underwent speciation to distinguish TB from non-tuberculosis mycobacteria (NTM) infections.

All household contacts under the age of 5 were referred for further clinical evaluation through routine services, including assessment of eligibility for isoniazid preventive therapy [17], according to South African guidelines [16]. Results from the laboratory tests were made available to study personnel, and all positive results were reported to participants and clinical staff for initiation of TB treatment through routine clinical services. Clinic records were evaluated to determine if those referred for treatment initiated anti-TB therapy. We did not provide HIV testing, but referred those who did not know their HIV status, or who had not been recently tested, to the routine health services for voluntary HIV counseling and testing.

If household contacts were not available for recruitment during the first study visit, study staff attempted to make an appointment to return to the home at a later time. Study staff returned to each household up to three times to complete recruitment and deliver all positive test results. If a phone number had been provided and participants consented to receiving their results via phone, the study team called all those who had negative smear and culture results.

### Statistical analysis

Our primary outcome was the proportion of household contacts with newly diagnosed TB, confirmed by smear or culture. We calculated the yield of contact tracing as the number of newly diagnosed TB cases identified per 100 index cases traced. We also converted the yield into the number of index case households needed to screen (NHNS), and the number of contacts needed to screen (NCNS), to identify one additional confirmed TB case. We constructed 95% confidence intervals (95% CI) around these estimates by assuming a binomial distribution (for prevalence) or a Poisson distribution (for yield, NHNS, and NCNS). We examined univariate associations between our outcomes and potential predictors using Fisher’s exact tests for categorical variables and Wilcoxon/Mann-Whitney tests for continuous variables. We used multilevel logistic regression to examine relationships between newly diagnosed TB and variables including demographics, laboratory results, symptom history, and index case characteristics, incorporating a random effects term to account for clustering at the household level. All analyses were performed in Stata 12 (Stata Corp., College Station, USA).

### Ethical considerations

The study was approved by the University of the Witwatersrand’s Human Research Ethics Committee, the Johns Hopkins Bloomberg School of Public Health Internal Review Board, and the Limpopo Provincial Government Department of Health. Study participants provided individual, written informed consent for inclusion in the study. Parental consent was obtained for participants less than 18 years of age; children ages 7–17 also provided assent.

## Results

### Index cases

From December 1, 2013 – September 30, 2014, we recruited 130 of 156 (83%) of eligible index cases from 27 participating public clinics in Vhembe District (Fig. 1a). Index cases were 56% male (73/130) and averaged 40 years of age (Interquartile range [IQR]: 31–49) (Table 1). Nearly all participants spoke the local language (Tshivenda) primarily (126/130, 97%) and were born in Limpopo Province (127/130, 98%). Just over half of index cases had completed at least some high school (73/130, 56%), and a similar proportion reported living in a female-headed household (67/130, 52%). On average, household size was five people, including the index case (IQR: 3–6), and the head of the household earned a median 2200 Rand (about US$200 in 2014) per month (IQR: R13-R2350) from all formal and informal sources. Index cases had lived in their current homes for a median time of 30 years (IQR: 20–40); only two index cases reported living in their current home for 1 year or less.

**Table 1:** Index Case Demographic and Clinical Characteristics

Variable	Overall (*N* = 130)	New TB in Household^a^ (N = 9)	No New TB in Household^b^ (*N* = 121)	*P*-value*
	N (%)	N (%)	N (%)	
Female Sex	57 (44%)	3 (33%)	54 (45%)	0.731
Age (Median, IQR***)	40 (31–49)	36 (31–48)	39 (31–49)	0.831
Female-Headed Household	67 (52%)	7 (78%)	60 (50%)	0.166
Head of HH Income (Median, IQR)	1270 (13–2350)	2500 (1270–6300)	1270 (0–2000)	0.047
Education				
8th grade or less	40 (31%)	4 (44%)	36 (30%)	0.278
At least some high school	73 (56%)	3 (33%)	70 (58%)	
More than high school	17 (13%)	2 (22%)	15 (12%)	
Unemployed	73 (56%)	6 (67%)	67 (55%)	0.731
Number of HH members, by Self-Report (Median, IQR)	5 (3–6)	6 (5–6)	4 (3–6)	0.130
Number of HH Contacts Participating in Study (Median, IQR)	2 (1–3)	3 (2–6)	2 (1–3)	0.005
Years lived in HH (Median, IQR)	30 (20–40)	37 (28–43)	30 (19–39)	0.235
Previous TB	15 (12%)	1 (11%)	14 (12%)	1.0
TB Symptoms				
Cough	58 (45%)	4 (44%)	54 (45%)	1.0
Fever	45 (35%)	1 (11%)	44 (36%)	0.162
Fatigue	64 (49%)	4 (44%)	60 (50%)	1.0
Loss of appetite	25 (19%)	1 (11%)	24 (20%)	1.0
Weight loss	84 (65%)	5 (56%)	79 (65%)	0.720
Night sweats	55 (42%)	5 (56%)	50 (41%)	0.493
At least one TB symptom	112 (86%)	7 (78%)	105 (87%)	0.611
Symptom duration				
No symptoms	18 (14%)	2 (22%)	16 (13%)	0.357
< 1 month	57 (44%)	3 (33%)	54 (45%)	
1–6 months	32 (25%)	1 (11%)	31 (26%)	
> 6 months	23 (18%)	3 (33%)	20 (17%)	
Smear Positive (overall *n* = 60)**	44 (73%)	2 (29%)	42 (79%)	0.012
Xpert MTB/RIF Positive (overall *n* = 64)^c^	55 (86%)	2 (67%)	53 (87%)	0.370

*Categorical variables were tested using Fisher’s Exact Test, and continuous variables were tested with the non-parametric Mann-Whitney test

**70 individuals were missing smear results in the clinic TB register and/or index case TB card (68 index cases living in households with no new TB cases, and 2 individuals living in households with ≥1 new TB case)

***IQR: Interquartile range

^a^Index cases from households with at least one member diagnosed with laboratory confirmed TB as a part of this study

^b^Index cases from households with no members diagnosed with laboratory confirmed TB as a part of this study

^c^66 individuals were missing Xpert results in the clinic TB register and/or index case TB card (60 index cases living in household with no new TB cases, and 6 individuals living in households with ≥1 new TB case)

Of the 124 (95%) index cases with known HIV status, 58 (47%) were living with HIV. Less than 20% of HIV-infected index cases (*n* = 11) were taking antiretroviral therapy at the time of their TB diagnosis, while 71% (*n* = 40) received their HIV and TB test results within 30 days of each other. Only two index cases had documented drug-resistant TB. A great majority of index cases reported having TB symptoms (cough, fever, night sweats, weight loss, and/or fatigue) at diagnosis (112/130, 86%), with a median duration of symptoms of 30 days (IQR: 30–120).

### Household contacts

From 130 index case households visited, we recruited 282 household contacts (Fig. 1b). Household contacts were somewhat younger than index cases (median age: 26 years, IQR: 17–50, with 23% younger than 15 years old) and were more likely to be female (203/282, 72%) (Table 2). Among adult participants (> 18 years old), half had completed at least some high school (99/198, 50%). Of the 119 (42%) household contacts willing to disclose their HIV status, 22 (19%) reported that they were living with HIV, 20 (91%) of whom were receiving antiretroviral therapy. Three participants reported that they were currently receiving treatment for TB and were excluded from subsequent analyses. Only 4% of household contacts (12/279) were unable to produce sputum of a sufficient quantity for testing (12/279); these persons were analyzed as TB-negative.

**Table 2:** Household Contact Demographic and Clinical Information

Variable	Overall (*N* = 279)	Lab-Confirmed TB (*N* = 11)	No Lab-Confirmed TB (*N* = 268)	*P*-value*
	N (%)	N (%)	N (%)	
Female Sex	201 (72%)	10 (91%)	191 (71%)	0.301
Age Category				
Under 5	10 (4%)	0 (0%)	10 (4%)	
5–14	55 (20%)	2 (18%)	53 (20%)	0.943
15–39	112 (40%)	6 (55%)	106 (40%)	
40–64	66 (24%)	2 (18%)	64 (24%)	
65 and Older	36 (13%)	1 (9%)	35 (13%)	
Female-Headed Household	146 (52%)	9 (82%)	137 (51%)	0.063
Head of Household Income (Median, IQR**)	1270 (0–2500)	1270 (1270–6300)	1270 (0–2350)	0.101
Education				
8th grade or less	140 (50%)	3 (27%)	137 (51%)	0.079
At least some high school	114 (41%)	5 (45%)	109 (41%)	
More than high school	25 (9%)	3 (27%)	22 (8%)	
Unemployed	149 (53%)	8 (73%)	141 (53%)	0.229
Body Mass Index (Median, IQR)	24 (20–28)	21 (19–29)	24 (20–28)	0.888
Current Smoker	11 (4%)	0 (0%)	11 (4%)	0.493
Previous TB Diagnosis	29 (10%)	1 (9%)	28 (11%)	0.879
History of IPT	5 (2%)	0 (0%)	5 (2%)	0.658
Number of Household members (Median, IQR)	5 (4–7)	6 (5–8)	5 (4–7)	0.248
HIV Status				
HIV-Infected	19 (7%)	0 (0%)	19 (7%)	0.792
HIV-Uninfected	97 (35%)	3 (27%)	94 (35%)	
HIV Status Unknown	163 (58%)	8 (73%)	155 (58%)	
TB Symptoms				
Cough	41 (15%)	0 (0%)	41 (15%)	0.377
Fever	42 (15%)	1 (9%)	41 (15%)	1.0
Fatigue	40 (14%)	0 (0%)	40 (15%)	0.374
Loss of appetite	13 (5%)	1 (9%)	12 (4%)	0.414
Weight loss	38 (14%)	1 (9%)	37 (14%)	1.0
Night sweats	51 (18%)	1 (9%)	50 (19%)	0.695
At least one TB symptom	122 (44%)	2 (18%)	120 (45%)	0.120
Symptom duration				
No symptoms	157 (56%)	9 (82%)	148 (55%)	0.465
< 1 month	53 (19%)	1 (9%)	52 (19%)	
1–6 months	32 (11%)	1 (9%)	31 (12%)	
> 6 months	37 (13%)	0 (0%)	37 (14%)	
Smear Positive	1 (0.4%)	1 (9%)	–	**–**
Culture Positive	28 (11%)	10 (91%)	18 (8%)	–
MTB Culture Positive	10 (4%)	10 (91%)	–	–
Smear or MTB Culture Positive	11 (4%)	11 (100%)	–	–

*Categorical variables were tested using Fisher’s Exact Test, and continuous variables were tested with a Ranksum test

**IQR: Interquartile range

**Table 3:** Factors Associated with Newly Diagnosed TB among Household Contacts

Variable^a^	Unadjusted	Adjusted^b^
	OR (95% CI)	aOR (95% CI)
Female Sex	4.06 (0.49–33.71)	4.51 (0.54–37.65)
Age (per 10 year increase)	0.97 (0.72–1.30)	1.00 (0.71–1.41)
Female-headed household	4.48 (0.87–23.08)	5.19 (1.06–25.44)
Head of household income (Per 500 Rand)	1.06 (0.96–1.17)	1.03 (0.94–1.14)
Education		
8th grade or less	Ref	Ref
At least some high school	2.14 (0.49–9.40)	2.08 (0.47–9.19) ^c^
More than high school	6.05 (1.07 - 34.40)	(6.88 - 1.19 - 39.66)^c^
Unemployed	2.28 (0.56–9.25)	2.48 (0.62–9.96)
Number of HH members (Index case self-report)	1.12 (0.88–1.43)	1.14 (0.92–1.43)
TB Symptoms		
At least one TB symptom	0.26 (0.05–1.30)	0.29 (0.06–1.44)
Symptom duration		
No symptoms	Ref	Ref
< 1 month	0.32 (0.04–2.80)	0.35 (0.04–2.98)
> =1 month	0.22 (0.02–1.90	0.25 (0.03–2.13)

^a^All variables refer to the household contact unless otherwise indicated

^b^Multilevel logistic regression model adjusted for Education status

^c^Multilevel Logistic regression model adjusted results for education status after controlling for female head of household

### Yield and number of households needed to screen

The intervention identified 11 (3.9%) new cases of confirmed active TB, for a household contact prevalence of 3940 per 100,000 (95% CI: 1980-6940). Of these, only one (9%) was smear-positive; the rest were positive on culture alone. An additional 18 (6.5%) persons (6450 per 100,000, 95% CI: 3870-10,000) had cultures that were positive for non-TB mycobacteria. The household contact tracing intervention therefore yielded 8.5 previously undiagnosed TB cases (95% CI 4.2–15.1) for every 100 index cases traced, giving a number of households needed to screen of 12 (95% CI: 7–24), and a number of household contacts needed to screen of 25 (95% CI: 14–51) to identify one new case of previously undiagnosed TB. Yield ranged from 0% (among children under 5) to 4.4% (among household contacts over 13), though these differences did not achieve statistical significance (*p* = 0.67).

### Predictors of TB

Overall, 44% (*n* = 122) of participating household contacts reported at least one TB symptom; these included cough (15%), fever (15%), lethargy (14%), loss of appetite (5%), weight loss (14%), and night sweats (18%), with a median symptom duration of 75 days (IQR: 14–365) and mean of 272 days. Contacts newly diagnosed with bacteriologically-confirmed TB had a substantially lower prevalence of symptoms than those without TB, though this difference was not statistically significant (18% vs. 45%, *p* = 0.12). Only 29 contacts (24%) reporting seeking care for their symptoms; none of these household contacts had confirmed prevalent TB.

We detected no differences between contacts with and without TB in terms of BMI, smoking status, history of previous TB, and history of isoniazid preventive therapy (IPT) (Table 3). All of the household contacts diagnosed with TB had started TB treatment by the end of the study period. In a multilevel logistic regression model including both education and female-headed household status, both variables remained independent predictors of newly diagnosed TB (adjusted OR [aOR]: 5.2, 95% CI: 1.1–25.4, for female-headed household, aOR: 8.2, 95% CI: 1.5–46.2, for finishing high school versus having less than 8 years of education).

## Discussion

This study found a high prevalence (3.9%, 3940 per 100,000) of previously undiagnosed TB among household contacts of newly diagnosed TB patients, only one-third lower than that (6075 per 100,000) observed in a similar contact tracing study in a peri-urban area with nearly three times the background incidence of TB [13]. The yield in our study of 8.5 new TB cases for every 100 index cases traced was substantially lower than that observed in the peri-urban area, however, where household contact tracing yielded 23 new TB cases/100 index cases traced. Household contact tracing in a peri-urban setting also resulted in a NHNS of 4.3, compared to 12 in our rural setting. Some of this discrepancy may be attributable to higher participation rates and/or larger household sizes in the peri-urban setting, where an average of 4 persons participated per household, compared to only 2 participants/household in our rural setting.

The sensitivity of smear for culture-confirmed active TB in this population was less than 10%, and despite the high prevalence of TB symptoms among index cases, household contacts with TB were no more likely to report symptoms than those without TB. This analysis demonstrates that, even in rural settings, household contact tracing can feasibly identify cases of active TB, but symptom screening and sputum smear microscopy are unhelpful in identifying TB cases. Thus, for contact tracing to have a meaningful impact in such settings, more expensive procedures (such as performing mycobacterial culture or Xpert MTB/RIF on all contacts), or radiography using digital chest X-rays for screening, will likely be required. This may be especially important for sub-populations, such as people living with HIV, for which sputum microscopy performs particularly poorly, and among which we found no cases of confirmed TB in our study.

While the World Health Organization (WHO) recommends TB screening for the household contacts of newly diagnosed TB patients (because of their elevated risk of TB disease), they do not recommend a specific algorithm [18]. Instead WHO provides a range of potential algorithms, such as testing only those persons with any cough, a cough of more than 2 weeks, or the presence of any TB symptoms (e.g. cough, fever, weight loss, night sweats, or lethargy) [18]. Had we used this symptom screen to identify household contacts for further testing, we would have missed 9 (82%) of the 11 undiagnosed prevalent TB cases – none of whom reported a cough. Options, such as screening using digital chest X-ray, may be a cost-effective approach to TB case-finding among household contacts, and should be explored in future research.

The fact that the majority of new TB cases identified by this study were asymptomatic and smear-negative indicates that household contact tracing in rural areas may identify cases of TB early, before substantial secondary transmission or TB-related morbidity or mortality can occur. Virtually all index cases recruited for our study were symptomatic, whereas household contacts with TB were no more likely to report symptoms than their family members without active TB. These findings suggest that TB cases captured by active contact tracing interventions are different than the cases captured through passive case detection, and that the diagnostic tests and screening algorithms required to detect them differ as well. While 73% of index cases were smear-positive, only one of 11 household contacts with prevalent active TB was positive on smear.

We identified a very high prevalence of non-TB mycobacteria (NTM) among household contacts, about 1.5 times as high as the prevalence of culture-confirmed TB. Other studies from South Africa have also identified high rates of NTM infection [19, 20]. This result raises questions regarding the timing of TB treatment initiation among culture-positive persons identified through contact tracing. Speciation takes approximately 5 additional days [21] from the time a culture positive result is returned. Avoiding treatment delays for those with active TB is paramount, but preventing unnecessary treatment should be an important consideration given the poor positive predictive value of mycobacterial culture for TB (38%) in this population. Testing household contacts with Xpert MTB/Rif rather than culture would allow for more rapid TB diagnosis, avoiding unnecessary TB treatment for NTMs and preventing treatment delays associated with active TB.

This study has a number of important limitations. First, although we screened more than 280 household contacts, our sample size of TB cases was small, leaving us without power to detect modest but potentially important differences between those with and without TB. Because we recruited index cases with and without laboratory-confirmed TB, it is possible that some of the participating index cases did not have active TB disease. However, this study sought to explore the feasibility and effectiveness of household contact tracing under operational conditions in which TB is not always bacteriologically confirmed. Finally, due to budgetary constraints in this small, pilot study, we were unable to perform HIV testing, chest X-ray, or TB testing with Xpert MTB/RIF for household contacts, or to perform genotyping to demonstrate transmission between the index case and household TB cases identified by the study. Previous genotyping studies in South Africa have found that a sizable portion of presumed case-pairs within households had TB strains that were genetically distinct [22, 23], suggesting that household TB transmission may not be responsible for all TB cases identified during household contact tracing. Further studies of TB contact tracing in rural settings could seek to expand the sample size, include additional data on room-sharing and contact duration, evaluate novel diagnostic tools including Xpert MTB/Rif and digital chest X-ray, study the cost-effectiveness of active contact tracing in this setting, and elucidate the relationships between HIV and TB status among household contacts.

## Conclusion

Household contact tracing of newly diagnosed TB patients using culture in a rural South African setting feasibly detected a substantial number of people with previously undiagnosed TB, nearly all of whom were smear-negative. Symptom screening was not an effective strategy for identifying cases in the household. Household contact tracing is an important component of comprehensive strategies to end TB in rural high-burden settings, though the poor sensitivity of smear and symptom screening may substantially increase the resources required to uncover the substantial burden TB in this population.

## Abbreviations

BMI: Body mass index
HIV: Human immunodeficiency virus
IPT: Isoniazid preventive therapy
MGIT: Mycobacteria Growth Indicator Tube
MTB: *Mycobacterium tuberculosis*
NHNS: Number of households needed to screen
NTM: Non-tuberculosis mycobacterium
RIF: Rifampicin
TB: Tuberculosis
